# AGEISM IN AI: ROADMAPS TO RESEARCH AND INTERVENTION

**DOI:** 10.1093/geroni/igae098.1436

**Published:** 2024-12-31

**Authors:** Clara Berridge

**Affiliations:** Chair

## Abstract

Risks that AI applications pose to older adults have not been thoroughly examined or understood in concert with other markers of identity such as race and gender. Ageism is not centered in scholarship on AI or algorithmic harms, and yet conditions in which AI can reproduce or introduce ageism have been identified by researchers and organizations like the World Health Organization. A multi-pronged approach is needed to address the diverse ways ageism is reflected and amplified in AI, from exclusion and misrepresentation of older adults to failure to enable transparency. This symposium will describe a broad swath of critical interventions into AI ageism at multiple levels. To orient all to the topic, Dr. Stypinska will present her original AI ageism framework that identifies five forms in which ageism manifests. Dr. Gallistl will offer a set of research priorities for gerontological research that challenge us to advance conceptualizations of AI in gerontology and engage with the lived experiences of AI in the lives of older adults. Dr. Brewer will discuss how intersectionality affects older adults’ lived experiences and envisioned futures with conversational AI technologies. Dr. Berridge will discuss the importance of supporting older adults and people living with dementia to engage knowledgeably with AI tools used in care. Dr. Grigorovich will discuss the ethical implications that arise when location tracking technologies are used to track the movements and activities of persons living with dementia in nursing homes. Presenters will provide insights about needed research and approaches to doing this timely work.

